# Identifying price bubbles in copper market: Evidence from a GSADF test approach

**DOI:** 10.1371/journal.pone.0290983

**Published:** 2023-11-06

**Authors:** Yushan Peng, Menglin Ni, Xiaoying Wang

**Affiliations:** School of Business Administration, Shandong Women’s University, Jinan, China; Institute for Economic Forecasting, Romanian Academy, ROMANIA

## Abstract

This paper uses the test proposed by Generalized Supremum Augmented Dickey-Fuller to identify whether there are multiple bubbles in copper price. The empirical results show that base on market fundamentals, there are seven bubbles existed from January 1980 to March 2023. Through analyses, the first two bubbles can be explained by the demand from Japan by the industry concentration and persistent supply constraint. The third to sixth bubbles are mainly negatively impacted by the global financial crisis and growing demand of China. The last bubble is caused by the economic recovery from Covid-19. The logit regression has stated that aluminum price, copper production, all metals index and GDP have a positive impact on copper bubbles, while China’s copper imports and precious metals price negatively explains copper bubbles. The main contributions are the investigation of the copper price bubbles, its determinants and the different technique of GSADF to detect copper price bubbles. Furthermore, it provides helpful information for those investors to make reasonable investment decisions and thus, avoid potential price risk.

## 1. Introduction

Since the start of the 21st century, the world has experienced several difficulties, including the global financial crises from 2007 to 2008 and the epidemic of Covid-19 [1, 2]. The topic of financial and economic stability and sustainability has drawn great attentions in recent years [3]. Copper is proved to be vital in many aspects such as industries, markets and has become a sign impact factor on economy [4]. It is also an indispensable strategic resource for economic development and industrialization and the second largest strategic resource after oil. The electrical sector makes extensive use of copper. A significant factor influencing the growth of electromobility and the entire energy infrastructure is the potential market shortage of copper [5]. Moreover, the strong conductivity of copper, in particular, makes it a vital resource for advancing clean and renewable energy technology due to copper can make electrical equipment much more efficient [6]. As a result, copper is widely used in solar, wind, and hydropower systems [7]. Among China’s 124 existing industries, there are 113 industries which linked to the copper industry. Since the copper industry is located in the upstream of China’s industrial chain, the supply of copper products affects the downstream industries, especially the development of infrastructure and basic industries. Therefore, the identification of copper bubble can predict the development of copper price in advance, so as to ensure the safety of national economy and trade. Furthermore, it has been suggested that the demand for precious metals such as gold and copper has increased as a safe haven because of risk diversification [8]. Financial market participants such as banks and investment funds regard copper price as an important reference index in metal futures markets [9, 10]. In the past decades, international copper price has fluctuated frequently, and deviated from its fundamental value [11]. The recent research has demonstrated that the average annual price of copper reached its highest value ever in 2010, after the strong economic recovery from the global financial crisis (GFC) of 2007–2008 [12]. However, the copper prices depend on many major external factors, such as speculation, variations in demand and supply, the U.S. dollar exchange rate and global economic crises [13–15]. There has been reported the drastic fluctuations in international copper prices, especially when economic crisis or wars occur in the world, because of the copper strategic role in economies.

Copper has also been known as one of the most widely used metals as an important industrial commodity that moves with the economic cycle. The copper price movement can often predict economic activity [16]. The price of copper is highly responsive to global demand, particularly to shifts in demand from China, which is the largest consumer [17]. Market participants interpret any fluctuations in copper demand as a reflection of global production, leading to a diverse range of copper prices [18]. Copper price has become a key factor for Chile which has been recognized as the top copper producer and exporter of the world [19]. Etienne [20] has found evidence consistent with the assume that the liquidity of market might effect on the formation and evolution of bubbles. Thus, it is anticipated that a deeper understanding of the copper prices and financial markets will be useful to a variety of people, including forecasters, traders, foreign and domestic investors, financial regulators, and other monetary authorities.

Furthermore, global economic growth is the most important indicator of global economic development, and its impact on copper prices is clear. Since copper is the basic raw material, its consumption is closely related to manufacturing, so the growth of industrial production properly reflects current copper consumption. When the economy grows, copper demand increases, which drives up the price of copper. During the recession, copper demand shrank and prices fell. Since the U.S. is the world’s largest economy, its relevant government departments regularly release some economic indicators, which have a greater impact on the copper market. The market price regards the dollar as the primary currency. Amidst the worldwide economic downturn in 2008, the Federal Reserve adopted measures to alleviate the situation. The printing of money and the implementation of low interest rate stimulus initiatives cause capital to flow out of the United States, leading to an abundance of liquidity and a decrease in the value of the dollar. Investors should consider various strategies to efficiently broaden the range of their investment portfolios. Consequently, substantial sums of money have exited the United States and been allocated to foreign currency, petroleum, copper, and other resources.

This article attempts to analyze the rational bubbles in the market of international copper. When an investor is willing to pay a price higher than its intrinsic value for an asset, the rational bubble is contained in asset prices. Meanwhile, operators want to sell assets in a high-priced way, which makes it an equilibrium price at current high prices. As the most widely known definition of bubbles for reference, there are occurrences of bubble phenomenon if the asset prices are departed further from their fundamentals. The basic relationship between the rational bubbles and observe the asset pricing from the perspective of utility maximization and hypothesis embedded in standard pattern was proposed by Gurkaynark [21]. The principal part of the passage is to examine the rational bubbles in the background of the current value. The model has two constituent parts, one is “bubble” part, and another is the part of “market fundamental”, which anticipates the discounted value of future capital gains. Under the circumstance, rational bubbles are not inaccurate pricing effects but an integral part of asset prices. On the basis of the scholar Diba and Grossman [22, 23], if the price level is integrated but have a smooth difference or exist in cointegration, then no bubble exists. Great deals of competing tests exist different power characteristics or sizes. There is no need to reach an agreement on the results. If these tests show that there is a bubble, the correct explanation is that they indicate that there is some non-stationary factor in copper price. It may be a bubble, but it could also be that the hypothesis based on unobserved fundamentals is not valid. They also believe that although rejection of stationarity or cointegration conditions does not prove the existence of bubbles, failing to reject is proof of the nonexistence of bubbles. Evans [24] proves that when explosive bubbles show periodic rupture behavior in the sample, the traditional co-integration test cannot detect explosive bubbles. Therefore, many effective measures should be taken to check whether there are multiple bubbles in the copper market.

Therefore, this article attempts to make contributions in several aspects. The main goal of this research is to conduct an empirical examination of price bubbles in copper from January 1980 to February 2023. Copper has been demonstrated to be essential in numerous key economic industries, encompassing construction, transportation, communication, power production, and industrial equipment [25]. The high volatility of copper prices could cause bubbles and further disastrous consequences due to various factors. Thus, it is necessary to detect bubbles for various stakeholders, regulators and market participants to minimize the repercussions. Secondly, the research makes uses of extensive and latest sample period, which covers the most recent global events and explains the origins of the bubbles. According to the previous literature, few research has studies copper price bubbles within the period of Covid-19 where the copper price has been stated volatile during this period [26]. Thirdly, the main factors that determine copper price are mentioned, and this article uses logit regression to explain their function in the developments of bubbles. Therefore, it is undeniable that the evaluation of causes and consequences of bubble is necessary. The application of the Dickey-Fuller (SADF) and generalized extended Dickey-Fuller (GSADF) method in this paper contributes to the existing literature by examining potential bubbles in the global copper market. An additional way to make a contribution is by creating innovative strategies for dating. Furthermore, it is noteworthy that it employs a recursive procedure for determining the critical value of standard right-tail ADF statistics, as well as the initial occurrence and subsequent folding of cross-time events.

The findings have shown that copper price from 1980 to 2023 has seven bubbles. The economic growth, economic crisis, changes of copper demand and supply, the U.S. dollar depreciation disputes between workers and employees of copper mining enterprises and economic recovery from Covid-19 are the main causes of copper price bubbles. Furthermore, the logit regression has stated that aluminum price, copper production, all metals index and GDP have a positive impact on it, while China’s copper imports and precious metals price negatively explains copper bubbles. It offers helpful details on the root causes of the various stakeholders, which should be continuously monitored to reduce losses. This article consists of 6 parts. Section 2 reviews the current literature on copper price bubbles. Section 3 presents the methodology. Section 4 shows data and empirical results. Section 5 concludes and provide policy suggestions.

## 2. Literature review

Typically, bubbles come with significant price swings that surpass their intrinsic worth [27]. According to the theory of rational expectations, if investors acknowledge that they will incur a cost exceeding the asset’s intrinsic value, the asset’s price increases as they anticipate selling it at a higher price later on [28]. Blanchard and Watson [29] were the first to introduce the notion of a logical bubble. According to Brunnermeier [30], bubbles are typically characterized by a significant surge in asset prices, which is subsequently followed by a sudden decline. In international nonferrous metals market, there was a certain degree of price bubbles [31]. The production of copper from mining is contingent upon the profitability, which is influenced by the price due to an ongoing dispute between international corporations and the domestic authorities. The correlation between metal prices and macroeconomic factors is widely recognized [32]. When a serious oversupply or undersupply of concentrate exists and the copper price decrease, many mines could be shut down or close their operations [33].

Numerous prior researches have examined the primary factors influencing the worth of copper commodities and the causes of price surges. The researches on the factors influencing changes in copper prices could be mostly classified into two categories. First, the abundant and latest literature examines copper price changes from the perspective of financial factors. According to some researchers, fluctuations in copper prices may be influenced by changes in exchange rates. This is because the dollar is the currency used to settle copper and other commodities. When all other conditions remain the same, variations in the value of the dollar can affect the prices of staple commodities in the international market [34]. Furthermore, Cifarelli and Paladino [35] held the point that increases in market speculation could result in considerable adjustments to prices’ underlying value. Mutafoglu et al. [36] discovered a noteworthy association between the prices of valuable metals and the holdings of non-commercial entities. The authors Bohl et al. hold the idea that energy sectors were examined and it was shown that there was a significant increase in activity as a result of widespread market speculation driven by the desire for potential profits [37]. According to Figuerola-Ferretti & McCrorie [38], speculation plays a significant role in promoting bubble behavior due to the perception of copper as an investable asset. The findings of Su et al. The emergence and bursting of multiple copper price bubbles can be attributed to financial factors, including the depreciation of the U. S. dollar and speculative activities, which play a crucial role. Umar et al. [39] define a bubble cycle as a period of excessive activity followed by the bursting and collapse of asset bubbles so that the authorities must have powerful analytical tools to spot the start of a bubble and whether to intervene along the way. According to recent discoveries, financial elements play a more significant role in forecasting bubbles in the prices of precious metals, whereas the prediction of bubbles in metal prices requires the consideration of monetary policy rate and the production index [40]. BARTOŠ et al. [41] have shown that has indicated that the cost of copper is influenced by various factors, including supply and demand, the state of the global economy, the prevailing exchange rate of the dollar, and other elements that contribute to its volatility.

From a supply and demand standpoint, the second classification examines variations in copper prices. Based on traditional microeconomic theory, an imbalance between supply and demand ultimately results in price variation, impacting the inherent worth of nonferrous metals [42]. In his study, Humphreys [43] analyzed the metal price formation process, which transitioned from a significant increase to a sudden decline from 2003 to 2008. He concluded that the economic prosperity of the global economy can be considered the main catalyst for the remarkable surge in metal prices from 2004 to 2007. According to some experts, due to the low price elasticities of copper demand and supply, even minor shocks could lead to notable price surges and considerable instability [44]. Cheng et al. adopt a panel data vector autoregression (PVAR) approach, it was discovered that the volatility in metal prices within the international market is primarily influenced by enduring supply and demand factors [45]. Moreover, various authors have discussed additional factors that impact the price of metals, including fluctuations in oil prices [46], the ongoing Covid-19 pandemic [26], as well as earthquakes occurring in Chile and Peru [47].

The existence of financial market bubbles has been explored with several different techniques. Lucas [48] presents the asset valuation framework and serves as the foundation for examining rational bubbles that occur when asset prices diverge from fundamental values. In other relevant studies, various techniques for examining bubbles consist of the momentum threshold autoregressive (MTAR) model [49], as well as the Markov Switching Augmented Dickey-Fuller (MS ADF) test [50], among others. According to Evans [24], the unit root test is not effective in detecting periodically ruptured bubbles unless the probability of rupture is significant. The Supremum Augmented Dickey-Fuller technique is a formal statistical examination to determine the presence of bubbles, unlike other methods that rely on subjective assessments that deviate from fundamental principles or moderate conditions. The authors Phillips and others. According to [51], the technique proves to be highly efficient when there is only one occurrence of a bubble in the provided data. Nevertheless, if the duration of the sampling period is sufficiently extended, indications of numerous asset price bubbles become apparent. Identifying multiple bubbles with cyclical collapse in econometrics is considerably more challenging than detecting individual bubbles. This difficulty usually reduces the recognition ability of existing test mechanisms. Consequently, Phillips et al. show that the Generalized Supremum Augmented Dickey-Fuller (GSADF) test is available to examine and quantify bubble phenomena when there are multiple bubbles present. A number of researches have been done recently.

In contrast to previous literature, this article attempts to make contributions in several aspects. First, by accurately identifying multiple copper bubbles, this article has significant implications for both the formation and nature of copper bubble behaviors. According to Chen et al., the course of realized volatility in nonferrous metal markets is depicted, however, the discussion does not include the behaviors of the explosive copper bubble. This article examines the reasons behind copper bubbles, encompassing both financial and supply & demand factors. Although this topic has not received much attention in the literature, it holds significant importance for policymakers and stakeholders. Second, compared to previous literature, this article uses extensive research period. Previous research examining copper price bubbles [15, 40] did not consider the period of the Covid-19 pandemic. Thirdly, this article employs GSADF [51] to detect bubbles in a long period. Many studies on identifying market price bubbles have utilized the unit root test, potentially leading to issues with reliability [15]. Furthermore, numerous studies have identified instances of inflated prices through the utilization of Markov switching ADF, multiple-regime switching, Monte-Carlo simulations, the momentum threshold autoregressive test, and MTAR methods [52]. Hence, the SADF and GSADF methods provide suitable instruments with improved discriminatory capability to detect copper bubble patterns. The findings indicate that there were seven instances of bubble episodes observed during the study period. Additionally, the development of copper price bubbles is influenced by both financial and supply and demand factors, which act as driving forces.

## 3. Methodology

Analysis based on Phillips et al. [51] indicate that the performance of SADF and GSADF tests is superior to previous economic methods due to it captures any explosion that occurs in the entire sample, and ensure adequate observation results to achieve estimated efficiency. GSADF test is not a simple ex post detection technology, but an anticipated dating algorithm which can assist regulators in market monitoring through early warning diagnostic test. Ideally, such early warning systems need to have a low error detection rate to refrain from dispensable policy measures and a high positive detection rate to ensure the early and effective implementation of policies. Due to the fact that the GSADF test has many advantages, the check of multiple bubbles in the copper market has become meaningful and effective.

Due to its ability to cover a larger number of data sub-samples and its increased window flexibility, the GSADF test is anticipated to outperform the SADF test in identifying explosive behavior across multiple events. The GSADF examination is not a straightforward technology for post-testing, but rather an anticipated date algorithm that can aid regulators in market surveillance by means of an early warning diagnostic test. Detecting numerous bubbles in the aluminum market is both significant and efficient. The study of rational bubbles in relation to market fundamentals originates from the asset pricing model proposed by Lucas [48] in the literature. Most studies in econometrics focus on testing the presence of an explosive bubble. Tirole [53] proposed a theory stating that asset or commodity prices are determined by their intrinsic values and market fundamentals, leading to the well-known phenomenon of price bubbles. Gürkaynak [21] proposed the following equation as a well-known model for testing the inherent bubble.

$$P_{\text{t}}={\left(1+r_{f}\right)}^{-1}E_{t}({\text{V}}_{t+1}+{\text{U}}_{t+1})$$ (1)

where *P*_t_ is the copper price in the period t, r_*f*_ is the free-risk rate, *E*_t_ is the expectation, *V*_t+1_ is the returns in the period of t+1 and *U*_*t*+1_ represents the invisible component in the market.

$$P_{t}{}^{c}=\sum_{i=0}^{\infty }(\frac{1}{1+r_{f}}{)}^{i}E_{t}(V_{t+i}+U_{t+i})\;for\;\text{i}=0,1,2$$ (2)

where $P_{t}^{c}$ is the fundamental price of copper price, *V*_*t*+*i*_ is the dividend of copper in the period *t*+*i*. It shows the determinants in the fundamental price without a bubble.


$$D_{t}={\left(1+r_{f}\right)}^{-1}E_{t}(D_{t+1})$$ (3)

Which is the series of random variables that satisfies the homo-generous expectational equation.


$$P_{t}=P_{t}^{c}+D_{t}$$ (4)

The fundamental model has two parts, a market fundamental part and a bubble part. Eq (4) represents the general solution to Eq (1) as a sum of a market fundamentals constituent and a bubble constituent.

There is an assumption about *B*_t_ to determine the asset price. When *B*_*t*_ = 0, it represents that the value of bubbles is zero and the price is fundamental. If *B*_*t*_ ≠ 0, it can be seen that there are explosions and the price of bubbles is not zero.

In view of the explosive property of bubbles, Diba and Grossman [22] used a stationarity test which relies on the standard ADF test or Phillips-Perron test [51]. Phillips et al. [51] improve the SADF test can identify the bubbles not only in financial but also physical assets. Homm et al. [54] proved the SADF test to be effective identifying in cyclical collapsing behaviors and superior than other bubble tests. Phillips et al. [51] provide the SADF test follows:

$$P_{\text{t}}=aT^{-\beta }+\phi P_{t-1}+\varepsilon_{t}$$ (5)

Where a is a constant, *T* is the sample size, *ϕ* > 1/2, *ε* ~ *NID*(0, *σ*^2^), *ϕ* = 1. Eq (5) allows a random walk process. We can suppose that s_1_ is the starting date, s_2_ is the ending date and *s*_*w*_ is the window size and *s*_2_ = *s*_1_ + *s*_*w*_. The equation as follows:

$$\Delta P_{t}=\alpha_{s1,s2}+\beta_{s1,s2}P_{t-1}+\sum_{i=1}^{j}\eta_{s1,s2}^{i}\Delta P_{t-i}+\varepsilon_{t}$$ (6)

Where *P*_*t*-1_ is the asset price *ε* ~ *NID*(0, *σ*), *j* is the number of lag order which is determined by significance tests [55]. The null hypothesis of the unit root is *β* = 1. And alternative hypothesis is that *β* > 1, which shows that *P*_*t*-1_ is explosive. To overcome the restriction in detecting periodically collapsing bubbles of conventional unit root tests [24], the supreme of recursively determined ADF T-statistics is used by Phillips et al. [51]. The SADF test of a forward-expanding sample sequence is the evaluation of the repeating ADF model. Also it can test the hypothesis through the sup value of the corresponding ADF statistic time series. The range of window size *s*_*w*_ is from *s*_0_ to 1, 1 is the full sample size. The starting point *s*_1_ of the sample sequence is at 0, and *s*_2_ = *s*_1_ + *s*_*w*_. The $ADF_{0}^{S2}$ statistic is from 0 to *s*_2_ for a sample. The SADF statistic is defined as

$$SADF(s_{0})=\underset{s2\in [s0,1]}{\text{sup}}ADF_{0}^{S2}$$ (7)

SADF is very effective to distinguish a single bubble in the sample. However, if the sample period is too long to contain many bubbles, Phillips et al. [51, 56] demonstrate that SADF is ineffective for more than two bubbles, because in the SADF test there would be unreliable and misleading information from the existent bubbles. To overcome this weakness, the window widths are flexible in generalized sup ADF (GSADF) test by changing both starting and ending point of the recursion [51, 56]. So there are more sub-samples in the GSADF test. GSADF text is more efficient than SADF test in detecting more than two bubbles occur in long time series of the data.

**Fig 1:**
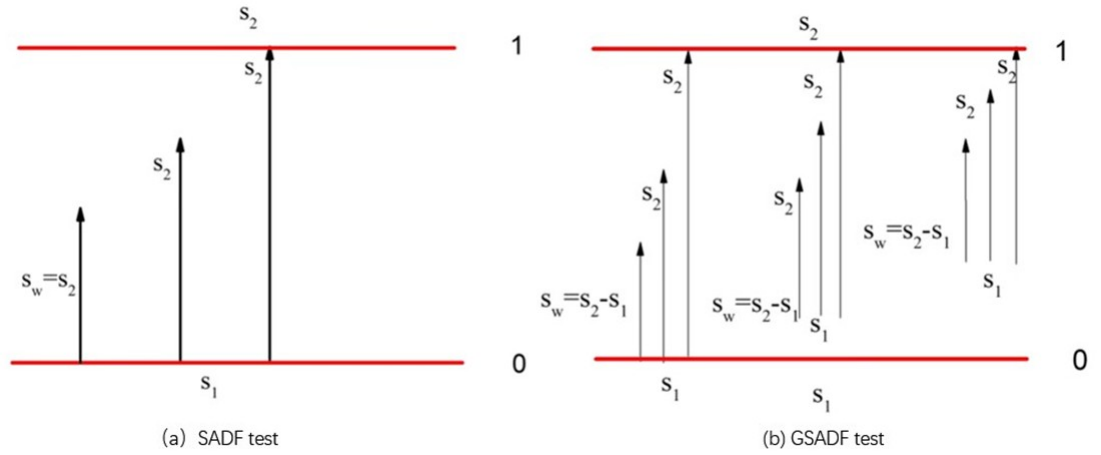
The sample sequences and window widths of the SADF test and the GSADF test.

The GSADF test is a repetition of the ADF test regression with a sample sequence data. But the sample sequence of GSADF is broader than that of SADF. GSADF allows the feasible starting points s_1_ to change within a little range from 0 to s_2_ − s_0_, and the ending point s_2_ varies from s_0_ to 1. Fig 1 illustrates the sample sequences of the SADF test and the GSADF test. So the GSADF test is better than the ADF test in detecting bubbles in long time episodes. The GSADF test is defined as to be the largest ADF statistic with the feasible ranges of s1 and s2 by Phillips et al. [51, 56], and denoted by GSADF (s0). That is,

$$GSADF\left(s_{0}\right)=sup_{s2\in [s0,1],s1\in [0,s2-s0]}(ADF_{s1}^{s2})$$ (8)

When there is an intercept in the regression model and the null hypothesis is a random walk, the limit distribution of GSADF statistic is:

$$ADF_{s1}^{s2}=\left\{\frac{(1/2)s_{w}[x{\left(s_{2}\right)}^{2}-x{\left(s_{1}\right)}^{2}-x_{w}]-\int_{s1}^{s2}x(s)ds[x(s2)-x(s1)]}{s_{w}^{1/2}{\left\{s_{w}\int_{s1}^{s2}x{\left(s\right)}^{2}dx-[\int_{s1}^{s2}x(s)ds{]}^{2}\right\}}^{1/2}}\right\}$$ (9)

Where *s*_*w*_ = *s*_2_ − *s*_1_ is a standard Wiener process. The SADF and GSADF statistics are according to the standard normal distribution in the condition of a random walk process. Phillips et al. [51, 56] simulation is applied to get the asymptotic critical values of the ADF statistic distributions with a random walk process. This standard Wiener process would be stochastic and sustainable, and only can generate a finite number of points. In this case, we can suppose that a Gaussian random in each finite interval equally spaced is created, and the intervals can be noted as *q*_1_, *q*_2…_*q*_*n*_. The right-tail critical values of GSADF are larger than those of SADF. Some simulations acquire the asymptotic critical values. To compute and recognize the bubble processes, a bootstrap methodology can be used to be effective proved by Monte-Carlo simulation.

Moreover, the BSADF statistic sequence is employed to ascertain the commencement and conclusion of every bubble. BSADF statistic is defined as:

$$BSADF\left(q_{0}\right)={\text{sup}}_{q_{1}\in \left(0,q_{2}-q_{0}\right)}\left\{ADF_{q_{1}}^{q_{2}}\right\}$$ (10)

The specified dates for the kth bubble’s appearance and end are as follows:

$${\tilde{q}}_{ks}={\text{inf}}_{{}_{q2\in \left(q_{0},1\right)}}\left\{q_{2}:BSADF\left(q_{0}>cv_{q_{2}}^{aT}\right)\right\}$$ (11)

$${\tilde{q}}_{k\text{e}}={\text{inf}}_{{}_{q2\in \left[{\tilde{q}}_{ks}+\delta log\left(T\right)/T,1\right]}}\left\{q_{2}:BSADF_{q_{2}}\left(q_{0}\right)<cv_{q_{2}}^{aT}\right\}$$ (12)

We evaluate the presence of numerous bubbles and determine the initial and final positions of these bubbles. This article explores the significant role of macroeconomic characteristics in copper bubbles, illustrated by Qt as follows:

$$Q_{t}=\left\{\begin{array}{ll} 0, & if\;BSADF_{q_{2}}\left(q_{0}\right)<cv_{q_{2}}^{aT}\\ 1, & if\;BSADF_{q_{2}}\left(q_{0}\right)<cv_{q_{2}}^{aT} \end{array}\right\}$$ (13)

where t = 1, 2……, T. When a bubble is noticed then Qt is equal to 1 and otherwise. The impact of macroeconomic factors on the copper bubble Qt is estimated by employing logit regression. We value it as below;

$$Q_{t}=E_{t}\alpha +u_{t}$$ (14)

where Et is the macroeconomic determinants of CP like China’s copper imports, GDP, aluminum price, copper production and alternative metal prices. The logit model is. described as

$$P\left(Q_{t}=1|E_{t}\right)=\phi \left(E_{t}\alpha \right)$$ (15)

The parameters are a mean of cause-effect which is estimated by the log-likelihood function and is defined as:

$$ln\;L=\sum_{t=1}^{T}Q_{t}\;ln\left[\phi \left(E_{t}\alpha \right)\right]+\sum_{t=1}^{T}\left(1-Q_{t}\right)ln\left[1-\phi \left(E_{t}\alpha \right)\right]$$ (16)

However, the marginal effect offers evidence about the degree of influence.


$$\frac{\partial p\left(Q_{t}=1|E_{t}\right)}{\partial E_{j}}=\phi \left(E_{t}\alpha \right).\alpha_{j}$$ (17)

## 4. Data and empirical results

In order to test the presence of bubbles in the copper market, we used monthly data from January 1980 to March 2023. These data are from the International Monetary Fund database (IMF), which has been open to the public since January 1980. The LME is the world’s largest non-ferrous metal exchange, trading in copper, aluminum, lead, zinc, nickel and aluminum alloy. The price and inventory of the exchange have an important impact on the production and sales of non-ferrous metals around the world. And the data derives from London Metals Exchange (LME) spot price of grade ‘A’ cathode obtained from the LME (CIF European ports). In the sample period with financial, geopolitical, speculative events and crisis. In the global copper markets, LME is used in copper spot and long-term contracts. Furthermore, the LME copper spot price is widely accepted as one of the main international benchmarks [13].

Relatively stable copper prices and their volatility ranges from $2592.63 to $8856.31 per metric tons from January 1980 to March 2023. In the early 1990s, when western countries entered a new round of economic weakness, copper prices fell from $2,969 per ton in 1989 to $1,995 per ton in 1993. Since 1994, western economies such as the U.S. began to recover, demand for copper increased and prices began to climb again. In 1997, when the Asian economic crisis broke out, the copper consumption in the whole Asia (except China) fell sharply, leading to the continuous decline of copper price to the lowest level in 20 years. On the contrary, in the second half of 1999, the Asian economy improved and copper prices gradually recovered. During 2001 to 2002, the copper market was dominated by world economic trends. The U.S. economy suffered a downturn, and the economies of major western countries were also greatly affected. In 2003 and early 2004, the U.S. economy was recovering and, more importantly, China’s rapid economic growth had created a surge in demand for copper that sent prices soaring to $3,000. When experiencing prices were ultra-low, copper prices rose sharply from $1272.07 to $ 3117.33 per metric ton, peaking with appearing in April 2009, from $2728.461 to $ 9880.938 per metric ton, peaking with appearing in February 2011, and the rising range fourth time of the original price. Wen et al. [57]argue that copper supply cannot match growing demand, especially in China, where two leading steeply rising spot prices for copper price. Since then, the global copper market has experienced the worst decline in demand under the impact of the global financial crisis in 2008, with prices falling to $3105.1 per metric ton in December 2008. As U.S. Congress $800 billion and China’s ¥4 trillion economic stimulus policy in 2009, copper prices are rising again. When it comes to 2020, Covid-19 pandemic has deeply influenced global economy. The demanding of copper has inevitable influenced by the shutdown of manufacturing industries. The price of copper has experienced a short period of decline which ranges from $6077.06 in December 2019 to $5057.97 April 2020. Soon after the shock of first wave of Covid-19, the copper price began to rise again.

The SADF and GSADF test are used to identify the bubble periods in copper market with Monte Carlo simulations through 10,000 replications. Some conclusions could be shown in Table 1. Based on the process of analysis, we conclude that there are copper bubbles in the global copper market. The SADF statistic is 5.959 and GSADF statistic is 8.524 for the full data series. Respectively these overstepped 1% right-tail critical value (e.g. 5.959 > 2.026, 8.524 > 2.818). So the null hypothesis of *H*_0_: *β* = 1 is rejected which shows that there are explosive sub-periods in the price of copper. Therefore, through the SADF and GSADF tests the conclusion shows that there is exuberance in the price of copper, and we can identify possible presence of bubbles.

**Table 1:** Results of the SADF and GSADF tests.

Copper price	SADF	GSADF
	5.959***	8.524***
Critical value		
90%	1.129	1.973
95%	1.440	2.221
99%	2.026	2.818

Note:

***denotes significance at 1% level

**Fig 2:**
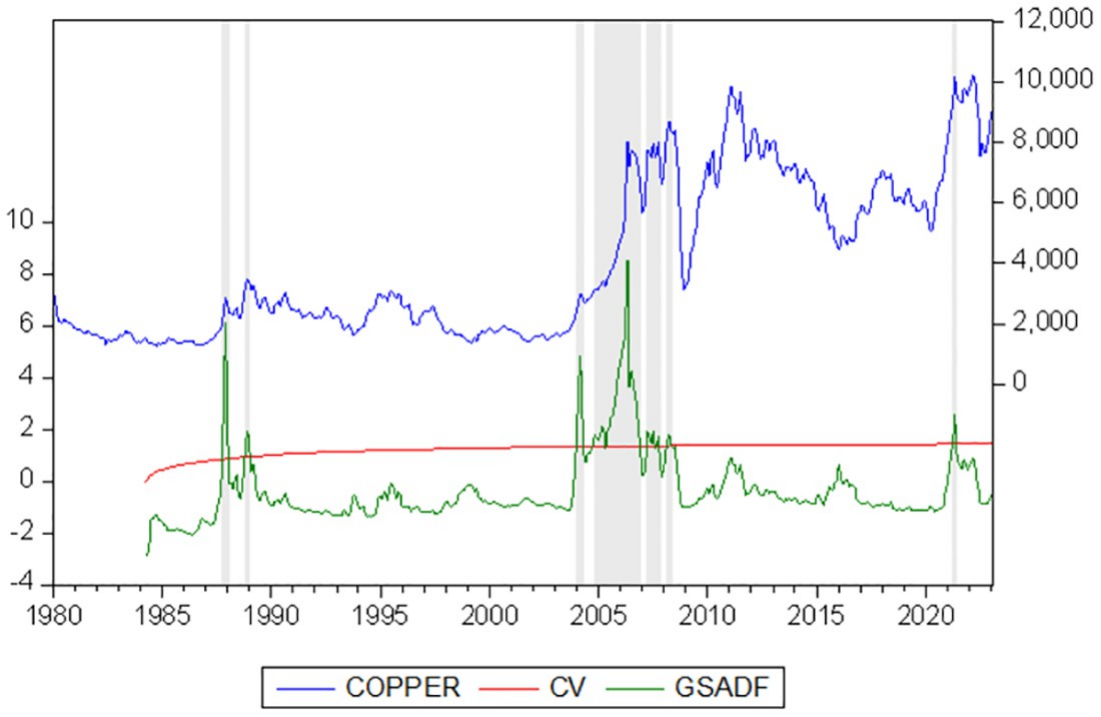
GSADF test of the price of copper. Note: the shadows are sub-periods with bubbles.

Base on the GSADF tests results, the estimate of the copper price can be graphed as Fig 2. The lower curve stands for the GSADF statistic. The middle curve represents the 95% critical value. The upper curve is the price of copper. Concerning the generation and burst of bubbles, there are seven bubbles during in the full sample period. The GSADF test has been certified to outperform SADF by Phillips et al. [56], because the GSADF test can broaden sample size with the flexible window widths in detecting explosive behavior and seldom giving false results. And the GSADF test could cover more data subsamples. Based on this, we can further identify the multiple bubbles and causes in the copper markets.

Table 2 provides additional insights into the duration and extent of seven instances of bubble phenomena observed in the global copper price. Notably, the period spanning from 2004 to 2008 encompasses the lengthiest bubble episode, lasting for a duration of 26 months. Furthermore, it can be inferred that these seven bubble occurrences coincided with significant fluctuations in the copper market prices. Specifically, the commencement of the longest bubble episode coincided with a substantial price change of 157.457%. Additionally, the phenomenon of copper bubbles tends to manifest during periods of significant price escalation, subsequently deflating abruptly when prices experience a sharp decline.

**Table 2:** Bubble length and price changes during bubble episodes.

Bubble periods	Length (month)	Start to peak%	Peak to end
1987M10-1988M02	5	31.385	-18.769
1988M11-1989M01	3	5.875	-2.964
2004M01-2004M05	5	23.891	-9.06
2004M11-2006M12	26	157.457	-17.101
2007M04-2007M11	8	4.272	-14.028
2008M03-2008M05	3	3.318	-4.109
2021M04-2021M06	3	9.024	-5.26

Through Fig 2 can be seen that the first two bubbles appeared in October 1987 to February 1988 and November 1988 to January 1989. The copper price increased from $1966.52 to $3392.9 per metric ton, an increase of 72.5%. The most important event on copper markets is rapid economic development in Japan, which has been little affected by the second oil crisis. This led to a large increase in copper demand, because of the intensity of metal use with a country’s dependence on its economic development [58]. At that time, Japan’s economic indicators reached an unprecedented high level. However, as the rising asset prices could not get support from industry, the Japanese government felt pressure and the Bank of Japan decided to change the direction of monetary policy. The Bank of Japan has raised the central bank discount rate five times from 2.5% to 6%. The Japanese economy began to decline in 1989, and the crisis stood out to be particularly severe [59]. Meanwhile, the worst economic crisis occurred in Latin America after the Great Depression, and average inflation reached to the level of nearly 1,000% around 1988 [60]. Due to Latin America is the largest copper producer, then the crisis has inevitably affected the copper market. With the economic recession in Japan and the economic crisis in Latin America, investors hold negative expectations for the future economy and fled a large amount of money from the commodity market. The relationship between supply and demand changed and demand further shrank, which further pushed down the international copper price. Finally, the continued market recession led to the bubble burst.

The third bubble began in January 2004 and lasted four months to May 2004. The next three bubbles are found between November 2004 to December 2006, April 2007-November 2007, and March 2008 to May 2008. One of the bubbles lasts longer than ever, which is more than two years. During 2004 to 2008, the copper price dynamics was fluctuated in the range of $2421.48 to $8714.18 per metric ton as high volatility, which suffered from underlying fundamentals of copper markets and world economy. Firstly, copper prices are under pressure due to rigid copper supply and expanding world copper demand. Copper supply and demand in 2004 had been tightening. According to the research and statistics of Swedish raw materials group, global refined copper output in 2004 was 15.826 million tons, with an annual growth rate of 3.3%. According to that, the statistical extractive electrode position method produced 2.627 million tons of copper, and the regenerated copper output was 5.482 million tons, up by 10.51% and 6.11% respectively. On the demand side, global consumption of refined copper reached 16.716 million tons in 2004, up 9.2% year-on-year, resulting in a global supply gap of 890,000 tons. Generally speaking, the asymmetry of supply and demand in copper market has promoted the rise of copper base price in this stage. The first reason for the bubble is global world demand for copper continued to rise sharply. After the war against Iraq, the Bush Administration’s tax proposals were approved as Growth Tax Relief Reconciliation Act (JGTRRA) of 2003. The principal feature of this legislation is reducing marginal tax rates which could improve the prospects for economic growth [61]. Secondly, the world economy began to recover in 2003. In 2004, world GDP grew by 5.05%, while China’s GDP grew by 10.1%, and in 2005, China’s GDP grew by 11.3%, which is the biggest producer and consumer of copper in the world [62]. In this period, there was the most important economic plan as the 11th national Five-Year program outlined in China, which included faster urbanizing and revitalizing the equipment manufacturing industry, and this accounted for the sharply increasing copper demand [63]. Meanwhile, the cable production grew 73.34% year-on-year in China. From June 2003 to August 2008, dramatic real estate price growth despite tightening monetary policy. During this period, the Chinese real estate market continued to soar, which was overheated. The national house price growth index has reached to over 10% during the fourth quarter of 2007 [64], and capital flows into real estate, the growth of copper demand was obvious.

However, regarding the copper supply, firstly with the rise of copper price, disputes between workers and employees of copper mining enterprises continue. The most representative strike was at CODELCO Copper Mine in Chile, which led directly to copper supply constraints in the world. Owned and operated by the Chilean government, CODELCO is the biggest producer of copper globally.In 2006, CODELCO had control over 20% of the global reserves, resulting in the production of 11% of the world’s copper. The company’s sales amounted to US$ 17 billion, with profits reaching US$ 9.2 billion (CODELCO, 2007). In 2007, the CTC initiated a widespread strike with the participation of 20,000 employees, aiming to exert pressure on CODELCO and the government. The strike, named ’Equal Pay for Equal Work’, resulted in a daily loss of US$ 10 million for the company [65]. The strikes like this in Latin America reduced the copper production. Considering these influencing factors, the cost of copper has surged from $2421.48 to $8714.18 per metric ton, hitting an all-time high with a remarkable rise of 245.1%. Furthermore, the devaluation of the American currency was sustained from the 1960s until the early 2000s. Consequently, this resulted in reduced investments in novel ventures, ultimately causing a rise in metal production [66]. The peak of copper prices is primarily attributed to the depreciation of the U.S. dollar, which is seen as the main driving force amidst tight supply and demand in the global copper market. In order to reverse the "double bankruptcy" situation, the U.S. authorities used lower interest rates and bond issuance. Many commodity markets, including the copper market, have experienced significant capital inflows and the formation of bubbles [67]. With an increasing number of investment funds aiming to make profits by holding positions in commodity futures contracts, there is consistently a higher quantity of call options compared to put options. This leads to an increase in the prices of futures and the prices of corresponding spot commodities [68]. Consequently, the cost of copper increased due to the impact.

The copper price bubble in 2004 was resulted from the changes in supply and demand. In 2004, the supply of copper became copper than before, which led to the impact of the copper market. After outbreak of American subprime crisis, the last bubble of copper burst. Under the copper accounting system denominated in U.S. dollars, the change of U.S. dollars has a significant impact on copper prices. The depreciation of the US dollar may be attributed to the subprime crisis in the U.S. capital market and the rescue policy of the US Congress [66]. Resource-dependent economies are experiencing the most severe decline in commodity prices during the transmission and impact of the global financial crisis worldwide.Oil and metal prices were hit hard by a significant price decline during the latter part of 2008 [69]. When the subprime crisis broke out in full swing, investors had negative expectations for the future economy, and commodity prices, including copper prices, fell sharply from $8714.18 to $3105.1 per metric ton. Another reason that account for the bubbles during this period is the price of oil. It has been reported that international copper futures prices follow oil prices due to the link between the prices of bulk commodities, while the relationship changes depending on the regime and is most favorable during the "steady rise" phase [46]. The oil price has been proved two bubbles occur during September 2004 to December 2004 and February 2008 to August 2008 which may account for the possibility of copper price bubbles. Therefore, with the recession of the capital market and the world economy, the bubble burst.

The last bubble appears in April 2021 to June 2021. The last bubble originated when copper price rising sharply due to robust global economic recovery from the impact of Covid-19. Covid-19 has been recognized as the greatest macroeconomic impact, which has had an impact on commerce, the global economy, and people in general [70]. According to WHO Coronavirus (COVID-19) Dashboard, the weekly deaths of Covid-19 reached that highest level in January and a dramatic decline since March 2021. Copper price has been witnessed a sharp rise from $8470.94 February 2021 and reached $10166.29 May 2021 per metric ton. The reason of the rise might be that copper, a crucial industrial metal used in production, is more likely to be in demand during economic expansion and crisis recovery [71]. Another factor that account for the fluctuation of copper price is that, Covid-19 pandemic has triggered uncertainty and volatility in commodity prices, investors are more likely to regard copper as safe haven due to risk diversification [8, 26]. It can be stated that the price of alternative metals could be one factor of the fluctuation of copper price. Therefore, the price of copper has experienced unexpected volatility during this period.

The above discussion explains several causal factors of seven bubbles. It can be concluded that the most significant determinants include China copper imports, GDP, aluminum price, copper production and alternative metal prices. Thus, in order to evaluate the underlying macroeconomic causes of CP’s explosive behavior, this article uses logit regression. The results are shown in Table 3. The log likelihood value greater than 50% critical value, demonstrating the strong explanatory power of the variables. A unit change in the independent variable causes a change in the dependent variable, which is explained by the marginal effect. The results show that these six factors are statistically significant. The China’s copper imports and precious metal price have had a negative effect on CP bubble. While the marginal effect suggests that the likelihood of a price bubble increases by 1.03% for a unit decrease in China’s copper imports and a price bubble increases by 19.61% for a unit decrease in precious metal price. It can be illustrated that investors tend to invest the higher price of precious metals, the more funds flow to precious metals, the less possibility of occurrence of price bubble. Meanwhile, aluminum price, copper production, all metal except gold index and China GDP have has a positive impact on copper price bubble. It has been illustrated by Bartoš et al. [41] that the demand for a replacement or alternative does not just arise from a lack of resources; according to economic theory, price rises will ultimately give way to lower-cost alternatives like replacements, new materials, or higher recycling rates. Thus, the aluminum price and all metal except gold index increases the demanding of copper will be higher and also increase the possibility of occurrence of copper bubbles. Additionally, the results also suggest that the impact of precious metals price is the greatest, followed by GDP, all metals except gold index, copper import, copper production is in the middle, and aluminum price is the least.

**Table 3:** Logit regression test.

Variables	Coefficients	Std. Error	z-Statistic	Marginal effect
AP	0.639231**	0.29	2.204	0.034
CP	1.536919**	0.717	2.143	0.082
CCI	-1.922080***	0.424	-4.534	-0.103
PMP	-8.018888***	1.701	-4.715	-1.961
AMP	3.449695***	0.846	4.078	0.844
GDP	4.631591***	1.229	3.768	1.133
constant	-4.544496***	0.544	-8.354	
Log likelihood	-93.80067			
LR statistic	154.4273			
Prob > Chi square	0			

## 5. Conclusions and policy suggestions

In this paper, we used the GSADF test proposed by Phillips et al. [56] to identify the existence of copper bubbles in the global copper market during 1980–2023. Overall, this paper shows that there are seven bubbles occurred in the international copper market in 1987, 1989, 2004, 2005, 2006, 2008 and 2021. Approximately over three-fourth of the copper bubbles have burst from January 2004 to November 2007. Also we consider the bubbles with the global financial crisis and other events. According to the empirical results, we can suppose that when the price is volatile the copper bubbles mostly appear. Generally, copper bubbles always occur during the period of price volatility, while bubbles affiliated by exploding demand, proportion of the supply and the global financial crisis lasts for a relatively short period. Identifying the starting and ending points of copper bubbles in the past years can recognize the significant symbols that cause the copper price to deviate from its intrinsic value. First, the sharp increase in global demand for copper, especially in Japan and China, has directly pushed up the price of copper. Therefore, Japan and China should improve energy efficiency and reduce the consumption of copper per unit output. Second, the supply-side industry concentration is relatively high. Chile, Peru and Zambia account for about 50% of international copper trade, suggesting that they can affect global copper market prices and achieve higher premiums. Therefore, the aluminum market should introduce more competitors. Finally, the global financial crisis has changed the monetary policies of various countries, especially the monetary policy of the U.S., which has had a significant impact on the world economy and led to asset bubbles.

Identifying the initial and final locations where copper bubbles have previously appeared could assist us in determining the key factors that cause the occurrence of copper bubbles. We contend that a surge in the copper market could potentially signify apprehension regarding an economic downturn among investors, serving as an initial indication of an impending financial catastrophe. Therefore, policymakers must be aware of the timing of copper bubbles as it can have significant consequences for financial stability. Moreover, copper has been viewed as the most traded basic metal used in several industrial applications. Copper price has also been shown in this article that will be inevitable affected by external variables and fluctuations. As a result, it makes the predictive models difficult. This article will provide solutions for governors and policymakers based on its empirical findings that might help the unstable copper market in terms of price stabilization while coping with economic crisis or pandemics like Covid-19. First, since this article provide evidence regarding the relationship between China’s copper imports, copper production and copper price bubbles. It is vital to stabilize the production and trade predictions of China’s copper industry and ensure the stable supply of copper production enterprises. Government should guide businesses in a reasonable manner in setting up their import and export trade schedules, stabilizing copper output, and avoiding centralized import growth or rapid import drop. Moreover, governments should promote the diversification of import or export channels and actively seek international cooperation. Second, global financial crisis has changed the monetary policies of various countries, especially the monetary policy of the U.S., which has had a significant impact on the world economy and led to asset bubbles. Therefore, the international copper pricing mechanism should take into account financial considerations in addition to fundamentals of supply and demand. Third, it is important for government to increase the technological innovation of copper products in emerging fields, further optimize the copper industry chain, and ensure the steady growth of copper consumption. Last, considering the impacts of Covid-19, strict remedial measures are needed, such as controlling economic activity by adhering to Standard Operating Procedures, which will reduce volatility in natural resource commodity prices via the industry channel.

## Supporting information

S1 Data (ZIP)
